# Participatory Online Surveillance as a Supplementary Tool to Sentinel Doctors for Influenza-Like Illness Surveillance in Italy

**DOI:** 10.1371/journal.pone.0169801

**Published:** 2017-01-11

**Authors:** Daniela Perrotta, Antonino Bella, Caterina Rizzo, Daniela Paolotti

**Affiliations:** 1 Computational Epidemiology Laboratory, ISI Foundation, Turin, Italy; 2 Centro Nazionale di Epidemiologia, Sorveglianza e Promozione della Salute, Istituto Superiore di Sanità, Rome, Italy; AOU Città della Salute e della Scienza di Torino, ITALY

## Abstract

The monitoring of seasonal influenza yearly epidemics remains one of the main activity of national syndromic surveillance systems. The development of internet-based surveillance tools has brought an innovative approach to seasonal influenza surveillance by directly involving self-selected volunteers among the general population reporting their health status on a weekly basis throughout the flu season. In this paper, we explore how Influweb, an internet-based monitoring system for influenza surveillance, deployed in Italy since 2008 has performed during three years from 2012 to 2015 in comparison with data collected during the same period by the Italian sentinel doctors surveillance system.

## Introduction

Influenza is an infectious disease caused by the Influenza viruses circulating during every fall/winter season in the temperate hemispheres and causing worldwide high morbidity and mortality epidemics with approximately 3 to 5 million cases and between 250,000 and 500,000 deaths [1]. Influenza surveillance is the ongoing, systematic collection, analysis, interpretation, and dissemination of data regarding the diffusion of the disease and the incidence for use in public health action to reduce morbidity and mortality and to improve health among the general population. The resulting data can be used for immediate public health action, program planning and evaluation, and formulating research hypotheses. Public health surveillance activities are generally carried out by public health officials. In particular for influenza surveillance, in most of the developed countries a national network of physicians traditionally report cases from individuals with influenza-like illness (ILI) and collect samples from a subset of patients for virological confirmation. In Italy, the sentinel surveillance system for influenza syndromes is called INFLUNET and it is coordinated by the Italian National Institute of Health, in collaboration with Centro Interuniversitario per la Ricerca sull’Influenza in Genova with the support of the Ministry of Health.

In recent years, advances in electronic data interchange and integration have increased the number and variety of systems allowing the collection of data from many diverse sources [2]. By leveraging on social and collaborative aspects of web technologies, a Web platform called Influweb (www.influweb.it), coordinated by ISI Foundation in Turin was deployed in Italy in 2008 and it has been active ever since [3]. The data collection is based on the self-reports of web volunteers, recruited among the general population, who are asked throughout the duration of the flu season to complete a weekly survey in order to report whether they manifested or not any respiratory symptom. This participatory form of surveillance provides epidemiological information directly from the general population, including individuals who do not seek health care treatment following an infection. Even though the sample of self-selected individuals is usually not representative of the general population [4], the information collected in a participatory fashion can help determine risk factors [5] and have a better overview of healthcare seeking behavior and vaccination effectiveness [6]. In this paper we want to evaluate the performance of this web-based participatory surveillance system in combination with the sentinel doctor surveillance as a tool for improving the national surveillance of Influenza-like illness. We also explore how participatory syndromic surveillance data can be used to estimate the burden of uncomplicated cases of influenza-like-illness in Italy. The integrated approach for flu surveillance has been explored since 2012, when a joint team formed by researchers of the National Centre of Epidemiology (CNESPS) of the Italian National Institute of Health and of ISI Foundation has undertaken the compilation of a weekly bulletin called FluNews [7] as a single collector of all information gathered by epidemiological surveillance systems monitoring the influenza in Italy [7], including the syndromic surveillance of access to Emergency Rooms and the monitoring of more severe influenza-related cases.

## Methods

The Italian sentinel surveillance system for influenza syndromes INFLUNET is coordinated by the Istituto Superiore di Sanità (ISS), in collaboration with Centro Interuniversitario per la Ricerca sull’Influenza (CIRI) in Genova with the support of the Ministry of Health. The system is based on a network of volunteering physician and pediatricians representing all the Italian regions. The participating physicians (about 1,000 each year) share a common operational protocol (http://www.iss.it/binary/iflu/cont/Prot16.pdf). The ISS analyses the data, inserted through a dedicated web site, and compiles a weekly report (http://www.iss.it/flue/index.php?lang=1&anno=2016&tipo=13). Thanks to the collected data, it is possible to estimate the weekly incidence of the influenza syndrome during the winter season to evaluate the duration and intensity of the influenza epidemic. Participating sentinel doctors are asked to report weekly influenza-like illness (ILI) cases, defined according to EU case definition (as mentioned in the protocol) occurring during the year, from week 42 to week 17. Specific information regarding age (0–4, 5–14, 15–64, >64 years) and influenza vaccine status are also collected and reported. Incidence rates are calculated using the number of ILI cases among the population under surveillance by age reported by each participating sentinel doctor. The system covers about 2% of the Italian population.

Influweb is an Italian internet-based cohort of self-selected participants followed over the influenza season every winter since 2008. It is part of a network of web platforms dedicated to participatory surveillance of flu since 2003 called Influenzanet (www.influenzanet.eu). Any individual living in Italy can be involved in the Influweb data collection by visiting and registering on the corresponding web site. Participation is voluntary and anonymous. The yearly study is disseminated among the general population at the beginning of each flu season through a number of press releases. Upon registration, users (i.e. individuals who have registered with the platform and possess a username and a password) are invited to complete an on-line one-time Intake Questionnaire containing demographic, medical and lifestyle questions. In particular, the Intake Questionnaire covers age, gender, household size and composition, location of home and workplace, education level, occupation, vaccination status for the previous and the present influenza season, the presence of a chronic disease, a possible pregnancy and other issues (see S1 File). Registered users are reminded weekly, via an e-mail newsletter, to fill in a Symptoms Questionnaire (see S1 File) in which they are presented a list of general, respiratory and gastrointestinal symptoms and asked whether they experienced any symptoms among those listed since the last time they visited the platform. Those who report any of the symptoms in the list are asked a series of follow-up questions, including the date of onset of the symptoms, the highest body temperature (if measured), if a fever was observed with or without sudden onset, whether and when they sought medical assistance, whether they took any medication, and whether they changed their daily routine. Users can also create accounts on behalf of other members of their family or household, thus enabling, for instance, parents to record data for their children.

In this paper, we have included data collected during the 2012–2013, 2013–2014 and 2014–2015 influenza seasons from week 47 to week 14 since for this time interval each year there is the full overlap of both systems for the data collection. In order to avoid having a variable and biased sample [8], mainly due to the possibility for volunteers to join the platform throughout the influenza season, for this study we have selected a subsample of users fulfilling the following characteristics:

- we have included participants who completed at least the Intake Questionnaire once since their registration date; if a user filled in multiple Intake surveys, we have used the most recently completed one;
- we have included participants who completed more than at least two Symptoms Questionnaires during the selected season with a frequency of at least one every three weeks, on average [9]–such individuals being referred to, hereafter, as active participants (in the following we will equally refer to Influweb population or Influweb active population to indicate the population of active participants).

Data from Influweb and Influnet have been compared with National Italian data sources: demographic data of the resident Italian population on 1^st^ January of 2013, 2014 and 2015 were provided by the National Institute for Statistics Studies (ISTAT), available at dati.istat.it; geographical data and shapefiles at the region level are publicly available at http://www.istat.it/it/archivio/24613. Georeferenced data from Influweb have been mapped from zip codes resolution to region level for comparison with national data. Summary statistics given here comprise simple counts and percentages. We used χ^2^-test for non-continuous variables, and non-parametric test (Mann-Whitney U test and Kruskal-Wallis test) to compare distributions.

Self-reported symptoms from Influweb active participants have been used to estimate the ILI incidence rates on a weekly basis during the three influenza seasons under study in order to understand whether the epidemiological signal in terms of number of ILI cases among the active participants has a correlation with the ILI incidence signal detected by the Influnet sentinel doctors surveillance. This has been done previously in other Influenzanet countries [10]. To estimate the ILI incidence among Influweb active participants, we have counted the number of active participants who each week reported symptoms corresponding to the ILI case definition given by European Center for Disease Control (http://ecdc.europa.eu/en/healthtopics/influenza/surveillance/Pages/influenza_case_definitions.aspx):

- Sudden onset of symptoms
- At least one of the following general symptoms: fever, chills, feeling tired, headache or muscle/joint pain
- At least one the following respiratory symptoms: sore throat, cough or shortness of breath

We included in the count only those active participants who reported their symptoms on a date within 15 days from the onset of the symptoms and, in order to avoid double-counting of a single ILI episode, we have discarded weekly ILI reports for individuals who have also reported an ILI episode during the previous week. In the few cases where individuals reported ILI for three consecutive weeks, we did not remove the third week from the data but only the second. The weekly incidence is calculated by dividing the number of ILI cases reported during a specific week by the number of active participants present in the cohort in that week. In order to compare more accurately the timing of the Influweb incidence curve with respect to the sentinel doctors incidence, we have analyzed the cross-correlation between the smoothed time series for Influweb data and the Influnet curve. The cross-correlation has been calculated with the cross-correlation function ccf() of the R stats package while the smoothing has been performed with the ma() function of the R package forecast. The ma() function computes a simple moving average smoother of the time series. We have used a time window of three weeks over which the average is performed.

Furthermore, self-reported symptoms from Influweb active participants and official surveillance data reported by Influnet have also been used to estimate age-specific influenza attack rates (the cumulative incidence of influenza virus infections). Similar studies have been carried out in The Netherlands [10] with data from the national Influenzanet platform called Grote Griepmeting and in the United States [11] with data from the Flu Near You platform. In this study, we have adopted the same methodology developed in Ref. 10, in which influenza attack rates are estimated through a combination of the Web-based participatory surveillance data on self-reported ILI (defined as self-reported fever and cough/sore throat) and a separate dataset, extracted from the Hong Kong household studies [12,13,14], consisting of symptoms reported by influenza-positive individuals in households where clinical cases of influenza had been observed.

We have used data on self-reported symptoms among active participants in the Influweb platform during the 2012–2013, 2013–2014 and 2014–2015 influenza seasons. For each season, we have defined four age-group-specific “main cohorts” (ages ≤ 24, 25–44, 45–64, and ≥ 65 years) as the set of persons in each age group who 1) had filled out a report by a “cutoff week”, that is the calendar week preceding the week in which the epidemic threshold is crossed and 2) number of completed reports during at least 50% of the weeks from the date of their first report through week 14. The cutoff weeks have been gathered from the official surveillance data reported by Influnet and correspond to weeks 2012–51, 2013–52 and 2014–51, respectively for the three influenza seasons.

The influenza attack rates, for which the mean and the 95% credible intervals are reported, are estimated by using the inference method described in Ref. 10, and, in particular, equation 3 in Ref. 10.

All the figures and statistical analyses were generated with IPython Notebook, version 3.0.0, and R, version 3.2.1. Maps were generated by manipulating the shapefiles of Italy at the region level with the Basemap library available in Python.

## Results

The number of participants to the Influweb project in the three seasons under examination has been quite steady. In total, 2,127 unique individuals participated during one or more of the three influenza seasons. In particular, 35.68% participated for one season, 22.99% for two seasons and 41.33% participated for all three seasons. The number of registered and active participants are reported in Table 1, as well as the number of symptoms surveys completed for each year.

**Table 1:** Participation to Influweb during the three seasons under study.

season	no. registered individuals	no. active participants	% active sample	no. active in country (per 100,000)	average symptoms surveys (95% CI)
2012–2013	3041	1452	47.75%	2.43%	10.4 (10.0, 10.7)
2013–2014	3487	1458	41.81%	2.4%	11.9 (11.5, 12.2)
2014–2015	3720	1464	39.35%	2.41%	12.4 (12.1, 12.7)

Referring to the 2014–2015 influenza season, among the sample of active participants, approximately 78% had a single membership account, while 22% belonged to a multiple account with at least two active participants. In particular, 11% of the multiple account had at least one participants aged less than 14 years and 9% had at least one participants aged over 65 years.

Among the sample of active participants, 49% of active participants never updated their Intake survey, 46% updated it twice, and 5% updated it at least three times during the 2014–2015 season.

**Fig 1:**
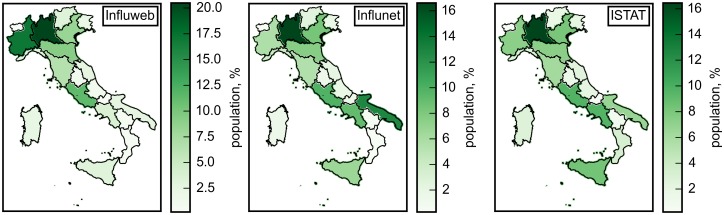
Geographic distribution of the Influweb active participants, the Influnet sample and the Italian population. The color code indicates the proportion of the population living in the Italian regions. The maps were created by manipulating publicly available shapefile at the region level.

During the three seasons in exam, the study has showed a coverage of all the regions in Italy, with an average of 2.41% active participants for 100,000. The active participation rate per region varies between 0.4 per 100,000 individuals (Calabria) to 5.61 per 100,000 individuals (Piemonte). The analysis of the geographical distributions at the region level of the Influweb active participants, the Influnet sample and the Italian general population, demonstrated that the three populations are not statistically different (*p* = 0.923 for 2014–2015, *p* = 0.925 for 2013–2014, *p* = 0.886 for 2012–2013). Fig 1 shows the three geographical distributions for the 2014–2015 influenza season only. The maps corresponding to the 2012–2013 and 2013–2014 influenza seasons are shown in Figures A and B in S2 file.

Table 2 shows the statistics about age, gender and household composition of the Influweb population compared to the Italian national data for the sample of season 2014–2015.

**Table 2:** Age, gender and household composition of the Influweb population compared to the general population.

season	age		gender		household size	
	Influweb, avg. age (95% CI)	IT population, avg. age	Influweb, %male, %female	IT population, %male, %female	Influweb, avg. household size (95% CI)	IT population, avg. household size
2012–2013	43.8 (42.9, 44.7)	43.5	57.8, 42.2	48.4, 51.6	2.6 (2.5, 2.7)	2.4
2013–2014	45.4 (44.4, 46.3)	43.7	59.1, 40.9	48.5, 51.5	2.8 (2.6, 2.9)	2.4
2014–2015	45.7 (44.8, 46.7)	43.9	58.2, 41.8	48.5, 51.5	2.9 (2.7, 3.1)	2.4

During the 2012–2013 influenza season participants were statistically representative of the Italian general population in terms of age (*p* = 0.226), whereas during the 2013–2014 and 2014–2015 influenza seasons participants were found to be older than the general population (*p*<10^−3^).

The comparison of the age distribution among the Influweb population, the Influnet sample and the Italian general population is shown in Figure C in S2 file. In the Influweb population the young adults and adults age groups are overrepresented, while school age children are underrepresented. On the other hand, in the Influnet sample, patients aged less than 25 years are slightly overrepresented, while adults aged between 25 and 64 years are slightly underrepresented.

**Fig 2:**
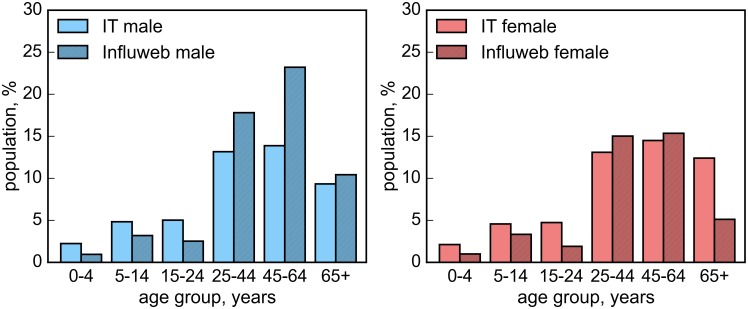
Influweb age and gender distributions. Age and gender distributions of the Influweb active participants in comparison with the Italian population for the 2014–2015 influenza season.

As shown in Table 2, a larger proportion of male participated to the Influweb project, in contrast with the gender distribution in the Italian general population (*p*<10^−4^ for all seasons). Fig 2 reports the age and gender distribution of the active participants in the Influweb sample compared to the Italian national data for the 2014–2015 influenza season. Similar results are shown in Figure D in S2 file for the 2012–2013 and 2013–2014 influenza seasons.

The distribution of the number of household’s members of Influweb participants is statistically different from the national one (*p* = 0.024 for 2012–2013, *p*<10^−4^ for 2013–2014 and 2014–2015).

Table 3 reports the vaccination coverage for the three seasons as reported by the Influweb active participants in comparison with the reports of the Influnet sentinel doctors. Vaccination coverage was statistically representative for the 2012–2013 (*p* = 0.722) and the 2013–2014 (*p* = 0.235) influenza seasons, whereas during the 2014–2015 Influweb detected a larger proportion of vaccinated people (*p* = 0.002).

**Table 3:** Influweb and Influnet vaccination coverage.

Season	Vaccination Coverage	
	Influweb	Influnet
2012–2013	15.15%	14.79%
2013–2014	16.46%	15.31%
2014–2015	16.19%	13.35%

**Fig 3:**
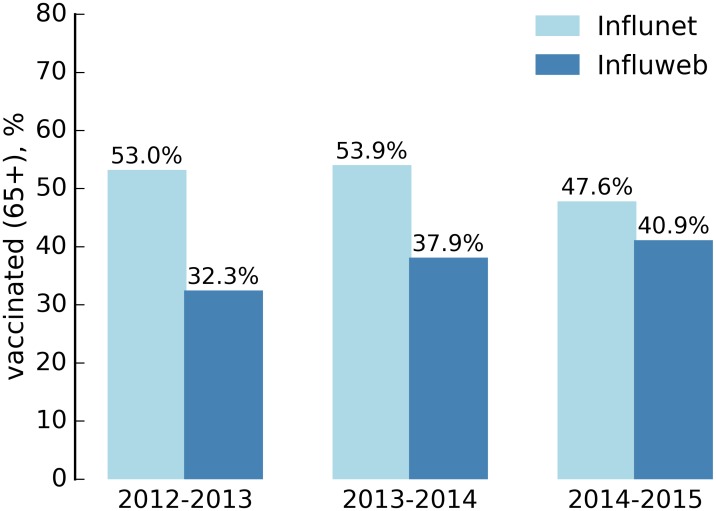
Influweb and Influnet vaccination rate for 65+ years old.

As shown in Fig 3, the vaccination coverage among people older than 65 years old was larger in the Influweb participants for the 2012–2013 and 2013–2014 influenza seasons (*p*<10^−4^), whereas it was statistically representative in the 2014–2015 (*p* = 0.06).

**Fig 4:**
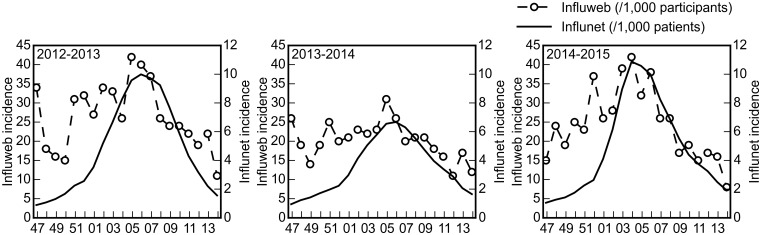
Incidence rates among the Influweb active participants in comparison with the Influnet incidence curve. For ease of comparison, the curves are reported on different scales.

The weekly incidence of ILI cases among Influweb users correlate well with the weekly incidence detected by the Influnet sentinel doctors for the three seasons under study. The Pearson correlation coefficient for 2012–2013 is 0.605 (*p* = 0.0047), for 2013–2014 is 0.472 (*p* = 0.0355) and for 2014–2015 is 0.699 (*p* = 0.0006). Fig 4 shows the weekly incidence curves of Influweb and Influnet, which are reported on different scales for ease of comparison. This is to highlight that the two curves are consistent in both timing and relative magnitude for the whole duration of each of the three influenza seasons under exam.

**Fig 5:**
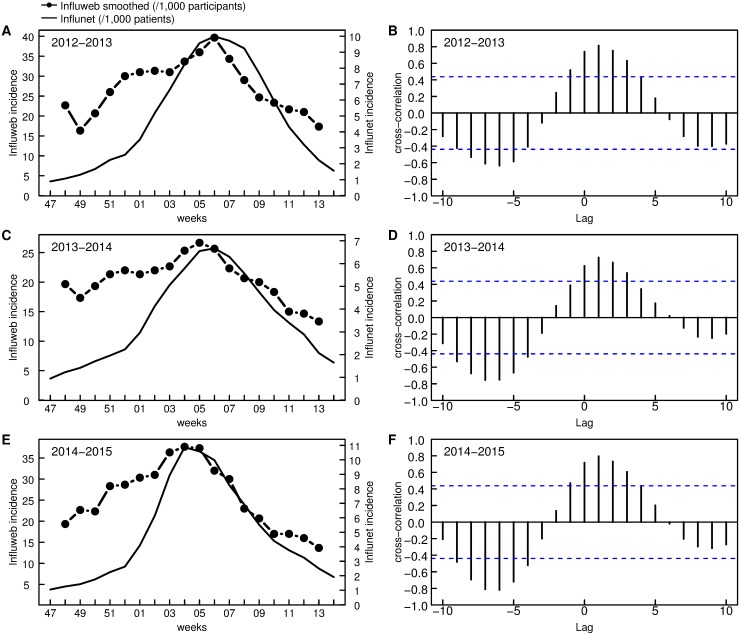
Cross-correlation between the smoothed time series for Influweb data and the Influnet curve. (A), (C), (E) show the incidence curves for Influnet and the smoothed one for Influweb. (B), (D), (F) show the cross-correlation as a function of the lag (weeks) between the two time series.

As shown in Fig 5, the analysis of the cross-correlation between the smoothed time series for Influweb data (3-weeks centered moving average) and the Influnet curve shows the maximum level of cross-correlation at lag of one week (ρ = 0.813 for 2012–2013, ρ = 0.724 for 2013–2014, ρ = 0.795 for 2014–2015).

**Fig 6:**
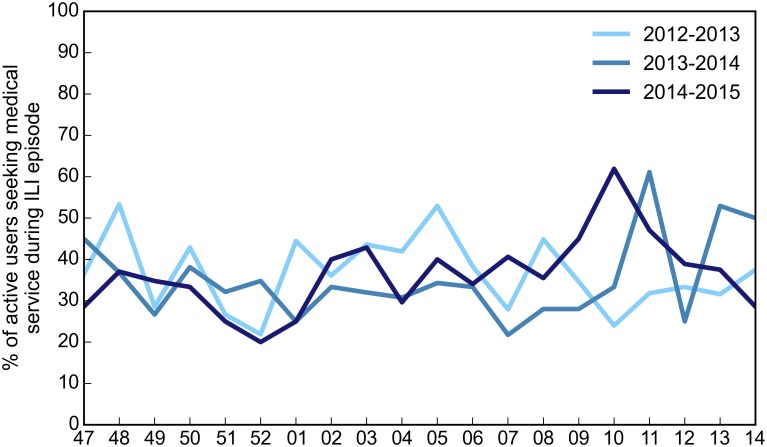
Health-care seeking behavior. Proportion of volunteers reporting ILI symptoms who sought healthcare assistance or consultation during the three influenza seasons under study.

**Fig 7:**
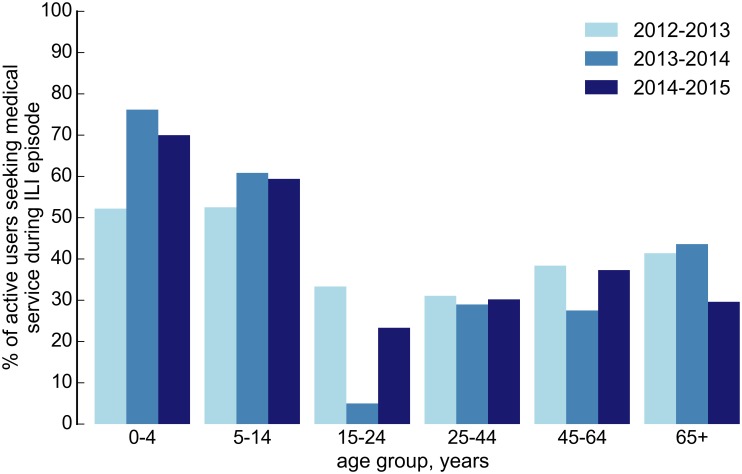
Age distribution of the proportion of participants seeking medical service during ILI episode.

As mentioned above, when reporting symptoms, participants were also asked to answer to some follow up questions regarding healthcare-seeking behavior and whether they changed their daily routine due to the illness. As shown in Fig 6 the weekly proportion of participants with ILI who reported visiting a sentinel doctor remained fairly constant during each season, with an average of 35.59%. The age distribution of the participants who reported a visit to their family doctor during an ILI episode is shown in Fig 7 and highlights the differences among age groups in healthcare consultation. In particular, the percentage of children for whom a healthcare provider has been consulted is significantly higher, even double for very young children, with respect to young adults.

**Fig 8:**
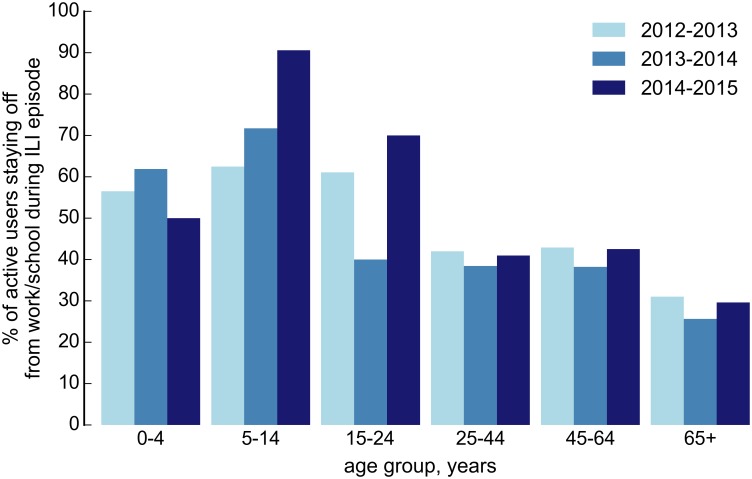
Age distribution of the proportion of participants who changed their daily routine during ILI episode.

Fig 8 shows the age distribution of participants who declared they changed their daily routine during an episode of ILI. Overall, children were more likely to stay at home from school, while elderly participants rarely reported that they changed their daily routine during an ILI episode.

In Table 4 we show estimates of influenza attack rates in the different Influweb cohorts during the 2012–2013, 2013–2014 and 2014–2015 seasons. We excluded from this specific analysis the age group of individuals who were younger that 24 years old because of the small size of the cohort for this age group. For the same reason, the 2012–2013 season, it was not possible to estimate an attack rate for the age group 65+ because of the small size of the sample. Sensitivity analysis with respect to the choice of the baseline period in equation 3 in Ref. [10] is presented in the supporting information (Table A in S2 file).

**Table 4:** Estimates of the Influenza Attack Rate in different age groups in Italy.

Season, Calendar Weeks	Estimated Attack Rate % (95% CI)		
	Age Groups, years		
	25–44	45–64	65+
2012–2013, 52–12	16.06 (0, 39.92)	18.72 (0, 38.95)	-
2013–2014, 01–12	10.75 (0, 31.3)	4.04 (0, 18.42)	12.04 (0, 28.4)
2014–2015, 52–13	16.2 (0, 41.39)	19.43 (0, 38.73)	15.32 (0, 38.72)

## Discussion

Participatory surveillance in Italy as a tool to collect cases of influenza-like illness among the general population has been implemented since 2008. In the past four years, data generated by the Web platform Influweb have been experimentally adopted by the Italian National Institute of Health as an additional source of data about the circulation of influenza-like illness among the population. The results presented in this paper are aimed at showing how the data collected by the web platform Influweb are reliable and useful as a support for the sentinel-based national surveillance in terms of providing information that are meant to enrich the existing methods for ILI surveillance.

A total of 2,127 persons actively participated during one or more of the three influenza seasons under examination. The number is low with respect to the number of individuals living in Italy (approximately 60 millions) but it has to be noted that the penetration of Internet usage among the Italian population is one of the lowest in Europe (http://www.internetworldstats.com/europa.htm). On the other hand, among the total active participants in the three seasons under study, approximately 38% participated for all three seasons, i.e. the sample is quite stable from one year to the other, with a consistent fraction of participants who continue to be motivated to participate over several years. The Influweb project has also been capable of attracting new participants, with an average of 1,458 active participants each season among which about 60% are new.

The geographical distribution of the Influweb active participants covers all the Italian regions and reflects well the heterogeneities of the population distribution in the various regions of Italy (see Fig 1). However, a higher number of active participants is consistently observed in the Piedmont region, which hosts the Institution conducting the Influweb project, likely reflecting a more powerful effect of communication campaigns at the local level.

The distribution of age is statistically different from the Italian general population. The young adults and adults age groups are overrepresented, while school age children are underrepresented. Underrepresentation in the groups between 0 and 25 years old may be due to the impossibility to access the Internet in an unsupervised way for the youngest children and to a lack of interest in influenza or health-related topics for teenagers and people in their 20’s, as pointed out in [7]. The system already incorporates the possibility of adding multiple users to an account managed by a single participant who is supposed to facilitate the input of data for individuals who cannot or are not familiar with Internet tools. The results for the 2014–2015 season showed that 11% of the multiple account had at least one participants aged less than 14 years and 9% had at least one participants aged over 65 years. It is interesting to note that elderly participants, i.e. the age group of individuals with more than 65 years old, is well represented. This might correspond to the fact that the familiarity with computers and the usage of the Web is increasing even among age groups that used to be considered harder to reach through the Web. It is also interesting to note that while for the Influweb sample there is an underrepresentation of children aged less than 25, this age group is well covered (if not slightly overrepresented) in the Influnet sample. This is a clear example of the kind of complementarity that the two systems can achieve.

The percentage of male participants is larger than the percentage of female participants, in contrast with the gender distribution in the Italian general population. The reason for this might be the fact that traditionally in Italy the familiarity with Web technologies is more typical of the male gender with a larger fraction of men (65%) accessing the Internet compared to women (55.8%), despite the fact that in general women are usually more active and involved on websites and forums dealing with health-related content [15]. This is also reflected on the very low rates of participation of elderly women.

Influweb participants live in larger households than the general population, in agreement with findings from other Influenzanet platforms [7].

Vaccination coverage is statistically representative of the national coverage in Italy during two seasons (2012–2013 and 2013–2014), whereas Influweb detected a larger vaccination coverage in Italy during the 2014–2015 influenza season. However, both systems have detected the slight decrease in the percentage of vaccination in the 2014–2015 influenza season. Looking at the 65+ age class, i.e. the target group for influenza vaccination and the age group who is most at risk of complications due to influenza-like illness, Influweb found a smaller proportion of vaccinated people in comparison with official data collected by the Ministry of Health [16] during the 2012–2013 and the 2013–2014 influenza seasons. This may be a consequence of the fact that this age class is not well represented in the Influweb cohort. On the other hand, as the sample size of this age group increased over the course of the years, in the 2014–2015 the vaccination coverage was statistically representative. Potentially, data from Influweb could also be used to estimate vaccine effectiveness during the influenza season [9].

As shown in Figs 4 and 5, the overall incidence rates calculated by means of Influweb data correlate well with those from Influnet sentinel doctors: the two curves are consistent in both timing and relative magnitude for the whole duration of each of the three influenza seasons under exam. This is in line with what has been observed previously with web-based platforms for influenza surveillance in other countries [17, 18]. Moreover, as shown in the panels B, D and F in Fig 5, Influweb detected the peak incidence one week earlier than sentinel doctors surveillance, thus suggesting that Influweb may be able to detect temporal variations in incidence rates in advance with respect to the sentinel doctors surveillance. This might be explained by the fact that most people generally do not seek healthcare assistance on the first day they feel sick, while sentinel doctors report the day of the visit as being the first day of illness, thus causing a slight delay in reporting.

The portion of volunteers seeking medical assistance when experiencing ILI remained fairly constant during each season, with an average of 35.59%. However, differences among age groups in healthcare consultation for individuals with ILI symptoms were evident, showing that children were more likely to be visited by a doctor with respect to young adults.

Most participants did not stay at home or changed their routine, when they experienced an episode of ILI. Overall, children were more likely to stay at home from school, as expected, while elderly participants rarely reported that they changed their daily routine during an ILI episode. This may be due to the fact that largest part of participants aged over 65 years were retired and only 9.3% still had a paid employment.

The syndromic surveillance data collected through the Influweb platform have also been used to calculate influenza attack rates during the 2012–2013, the 2013–2014 and the 2014–2015 seasons. The results show good agreement between results obtained for the weekly incidence of ILI in the Influweb population and attack rates measured by the inference framework described in the Methods. In fact, during the 2013–2014 season ILI attack rates were smaller compared to the other seasons, as the 2013–2014 influenza season was milder than the others. In the 2012–2013 season, it was not possible to estimate an attack rate for the age group 65+ because of the small size of the sample, but this shortcoming was fixed in the 2013–2014 and 2014–2015 seasons as the sample size increased. For the same reason, we restricted the estimation of influenza attack rates to the participants aged over 25 years, but future estimates can be performed even for the age group 0–24, provided that the corresponding cohort size would be sufficiently large. Overall, as already discussed in previous papers [10, 11], despite limitations of the inference method and of data gathered through the Influweb participatory system, the results show that it is possible to have real-time estimation of influenza attack rates among the general population during each influenza season. Such a feature is specific of the web surveillance systems detecting ILI cases directly from the general population.

## Conclusions

In the past four years, data generated by the web platform Influweb have been experimentally adopted by the Italian National Institute of Health as an additional source of data about the circulation of influenza-like illness among the general population. In this paper, we have presented the results of the three seasons during which the data from the participatory surveillance platform Influweb have been integrated with the official weekly bulletin compiled by the Italian national surveillance system in order to assess the contribution that an online system can provide to the traditional influenza-like illness surveillance systems. The results show that even though the sample of Influweb active participants is small with respect to the general population and not representative, the detailed information provided by the volunteers enables to estimate real-time weekly incidence and age-specific attack rates. Moreover, it is worth noting the possibility to account in the data collection for those individuals who do not seek health care attention when they experience an ILI episode

To improve the representativeness of the sample, targeted strategies for communication informed by the results of this paper can be used to increase participation rates in Italy, which are indeed lower than in other European countries in which Influenzanet is implemented. Nevertheless, the stability of the Influweb sample over the years guarantees in terms of detection of ILI symptoms a good epidemiological signal currently used to assess, in a multi-country fashion, the ILI activity during the flu season in the whole western Europe (https://www.influenzanet.eu/flu-activity/).

## Supporting Information

S1 File Intake survey and Symptoms survey (English versions). (DOCX)

S2 File Additional results. (DOCX)
