# Supplementary material for: Participatory Online Surveillance as a Supplementary Tool to Sentinel Doctors for Influenza-Like Illness Surveillance in Italy
Source: PLoS One. 2017 Jan 11;12(1):e0169801. doi: 10.1371/journal.pone.0169801 (PMC5226807; doi:10.1371/journal.pone.0169801)
Supplement: S1 File — (DOCX) [file pone.0169801.s001.docx]

## Intake questionnaire

**Intake Q0**

For whom are you filling this survey in? (If you are filling in the survey on behalf of someone else, then make sure that you have the consent of that person to do so)

- - Yes
  - No



**Intake Q1**
What is your gender?

- - Male
  - Female

**Intake Q2**
What is your date of birth (month and year)?

*Two drop-down lists*

**Intake Q3**

What is your home postal code?

XXXX /

**Intake Q4**
What is your main activity?

• Paid employment, full-time

• Paid employment, part-time

• Self-employed (businessman, farmer, tradesman, etc)

• Attending daycare/school/college/university

• Home-maker (e.g. housewife)

• Unemployed

• Long-term sick-leave or parental leave

• Retired

• Other



**Intake Q4b (If “Yes, Paid employment full time”, or “Yes, paid employment part time” to Q4 )**

What is the first part of your school/college/workplace postal code (where you spend the majority of your working/studying time)?

• XXXX

• I don’t know/can’t remember

• Not applicable (e.g. don’t have a fixed workplace)

**Intake Q4c (If “Yes, Paid employment full time”, or “Yes, paid employment part time” to Q4 Trigger free text if “other” checked, allow the users to write in their occupation )**

Which of the following descriptions most closely matches with your main occupation?

• Professional (e.g. manager, doctor, teacher, nurse, engineer)

• Office work (e.g. admin, finance assistant, receptionist, etc)

• Retail, sales, catering and hospitality and leisure (e.g. shop assistant, waiter, bar-staff, gym instructor etc)

• Skilled manual worker (e.g. mechanic, electrician, technician)

• Other manual work (e.g. cleaning, security, driver)

• Other


**Intake Q4d (to be asked for those aged 16 and over)**

What is the highest level of formal education qualification that you have? (If you are still in education, then please tick this box with the appropriate highest level that you have already achieved**)**

- I have no formal qualifications
- GCSE's, O’levels, CSEs or equivalent
- A-Levels or equivalent (e.g. Highers, NVQ Level3, BTEC)
- Batchelors Degree (BA, BSc) or equivalent (e.g. HND, NVQ Level 4)
- Higher Degree or equivalent (e.g. Masters Degree, PGCE, PhD, Medical Doctorate, Advanced Professional Awards)
- I am still in education



**Intake Q5**

Except people you meet on public contact, do you have contact with any of the following during the course of a typical day? (Select all options that apply, if any)

- More than 10 children or teenagers over the course of the day
- More than 10 people aged over 65 over the course of day
- Patients
- Groups of people (more than 10 individuals at any one time) oNone of the above



**Intake Q6**

INCLUDING YOU, how many people in each of the following age groups live in your household?

*Drop down menus for each of: 0-4 years 5-18 years 19-44 years 45-64 years 65+ years*

**Intake Q6b (If any in household are aged 0-18, including participant):**

How many of the children in your household go to school or day-care?

*Drop-down menu*



**Intake Q7**
What is your *main* means of transportation?

• Walking

• Bike

• Motorbike/scooter

• Car

• Public transportation (bus, train, tube, etc)

• Other

**Intake Q7b**

On a normal day, how much time do you spend on public transport (bus, train, tube, etc)?

• No time at all

• 0-30 minutes

• 30 minutes – 1.5 hours

• 1.5 hours – 4 hours

• Over 4 hours

**Intake Q8 (not a core question)**
How often do you have common colds or flu-like diseases?

• Never

• Once or twice a year

• Between 3 and 5 times a year

• Between 6 and 10 times a year

• More than 10 times a year

• I don’t know

**Intake Q9**

Have you received a flu vaccine this autumn/winter season (2012-2013)?

• Yes [go to questions Q10b and Q10c]

• No [go to question Q10d]

• I don’t know/can’t remember

**Intake Q9b: (if “Yes” to Intake Q10, follow-up question)**

When were you vaccinated against flu this season (2012-2013)?

• XX/XX/XXXX
• I don’t know/can’t remember

**Intake Q9c: (if “Yes” to Intake Q10, follow-up question)**

What were your reasons for getting a seasonal influenza vaccination this year? (Select all options that apply)

- I belong to a risk group (e.g. pregnant, over 65, underlying health condition, etc)
- Vaccination decreases my risk of getting influenza
- Vaccination decreases the risk of spreading influenza to others
- My doctor recommended it
- It was recommended in my workplace/school
- The vaccine was readily available and vaccine administration was convenient o The vaccine was free (no cost)
- I don’t want to miss work/school
- I always get the vaccine
- Other reason(s)

**Intake Q9d: (if “No” to Intake Q10, follow-up question)**

What were your reasons for NOT getting a seasonal influenza vaccination this year? (Select all options that apply)

- I am planning to be vaccinated, but haven’t been yet
- I haven’t been offered the vaccine
- I don't belong to a risk group
- It is better to build your own natural immunity against influenza
- I doubt that the influenza vaccine is effective
- Influenza is a minor illness
- I don’t think that I am likely to get influenza
- I believe that influenza vaccine can cause influenza
- I am worried that the vaccine is not safe or will cause illness or other adverse events
- I don’t like having vaccinations
- The vaccine is not readily available to me
- The vaccine is not free of charge
- No particular reason
- Although my doctor recommended a vaccine, I did not get one
- Other reason(s)

**Intake Q10**

Did you receive a flu vaccine during the last autumn/winter season?

• Yes

• No

• I don’t know/can’t remember

**Intake Q11**
Do you take regular medication for any of the following medical conditions? (Select all options that apply)

- No
- Asthma
- Diabetes
- Chronic lung disorder besides asthma e.g. COPD, emphysema, or other disorders that affect your breathing
- Heart disorder
- Kidney disorder
- An immunocompromising condition from treatment or illness including splenectomy, organ transplant, acquired immune deficiency, cancer treatment



**Intake Q12 (Only asked of women between ages 15 and 50)**

Are you currently pregnant?

• Yes [go to question Q13b]

• No

• Don’t know/would rather not answer

**Intake Q12b: (if “Yes” to Intake Q12, follow-up question)**

Which trimester of the pregnancy are you in?

• First trimester (week 1-12)

• Second trimester (week 13-28)

• Third trimester (week 29-delivery)

• Don’t know/would rather not answer



**Intake Q13**
Do you smoke tobacco?

• No

• Yes, occasionally

• Yes, daily, fewer than 10 times a day

• Yes, daily, 10 or more times a day

• Don’t know/would rather not answer

**Intake Q14**

Do you have one of the following allergies that can cause respiratory symptoms? (Select all options that apply)

- Hay fever
- Allergy against house dust mite
- Allergy against domestic animals or pets
- Other allergies that cause respiratory symptoms (e.g. sneezing, runny eyes)
- I do not have an allergy that causes respiratory symptoms



**Intake Q15 (not a core question)**

Do you follow a special diet? (Select all options that apply)

- No special diet
- Vegetarian
- Veganism
- Low-calorie
- Other

**Intake Q16 (not a core question)**

Do you have pets at home? (Select all options that apply)

- No
- Yes, one or more dogs
- Yes, one or more cats
- Yes, one or more birds
- Yes, one ore more other animals

## Symptoms questionnaire

If you are filling this in on behalf of someone else, please answer all the questions as if you are that person.

**Weekly Q1**

Have you had any of the following symptoms since your last visit (or in the past week, if this is your first visit)? (Select all options that apply)

- No symptoms
- Fever
- Chills
- Runny or blocked nose
- Sneezing
- Sore throat
- Cough
- Shortness of breath
- Headache
- Muscle/joint pain
- Chest pain
- Feeling tired or exhausted (malaise)
- Loss of appetite
- Coloured sputum/phlegm
- Watery, bloodshot eyes
- Nausea
- Vomiting
- Diarrhoea
- Stomach ache
- Other

**Weekly Q2** (If the participant was STILL ILL on their last visit and has reported symptoms this time):

On DATE OF LAST VISIT you reported that you were still ill with symptoms that began on DATE OF FIRST SYMPTOMS REPORTED PREVIOUSLY. Are the symptoms you reported today part of the same bout of illness?

• Yes

• No

• I don’t know/can’t remember

**Weekly Q3 (if symptoms)**
When did the first symptoms appear?

• Choose date XX/XX/XXXX

• I don’t know/can’t remember


**Weekly Q4 (if symptoms)**

When did your symptoms end?

• Choose date XX/XX/XXXX

• I don’t know/can’t remember

• I am still ill

**Weekly Q5 (if symptoms)**

Did your symptoms develop suddenly over a few hours?

• Yes

• No

• I don’t know/can’t remember


**Weekly Q6 (if fever)**
When did your fever begin?

• Choose date XX/XX/XXXX

• I don’t know/can’t remember

**Weekly Q6A (extra non-core question) (if fever)**

Did your fever develop suddenly over a few hours?

• Yes
• No
• Don't know

**Weekly Q6b (if symptoms)**
Did you take your temperature?

• Yes [go to Weekly Q6c]

• No

• I don’t know

**Weekly Q6c (if symptoms) and (if took temperature)**

What was your highest temperature measured?

• Below 37° C

• 37° - 37.4°C

• 37.5° - 37.9°C

• 38° – 38.9°C

• 39° - 39.9°C

• 40°C or more

- I don’t know/can’t remember

**Weekly Q7 (if symptoms)**

Because of your symptoms, did you VISIT (see face to face) any of medical services? (Select all options that apply)

- No
- GP or GP’s practice nurse
- Hospital admission
- Hospital accident & emergency department/out of hours service o Other medical services
- No, but I have an appointment scheduled

**Weekly Q7b (if symptoms)**

How soon after your symptoms appeared did you visit this medical service?

• Same day

- 1day
- 2days
- 3days
- 4days
- 5-7 days
- More than 7 days
- I don’t know/can’t remember

**Weekly Q8 (if symptoms)**

Because of your symptoms, did you contact via TELEPHONE or INTERNET any of the following?

(Select all options that apply)

- No
- GP – spoke to receptionist only
- GP – spoke to doctor or nurse
- Other

**Weekly Q8b (if symptoms)**

How soon after your symptoms appeared did you contact via telephone or internet any of the following?

• Same day

• 1day

• 2days

• 3days

• 4days

• 5-7 days

• More than 7 days

• I don’t know/can’t remember


**Weekly Q9 (if symptoms)**

Did you take medication for these symptoms?

- (Select all options that apply)
- No medication
- Pain killers (e.g. paracetamol, ibuprofen, aspirin, etc)
- Cough medication (e.g. expectorants)
- Antivirals (Tamiflu, Relenza)
- Antibiotics
- Other
- I don’t know/can’t remember

**Weekly Q9b (if antivirals were taken):**

How long after the beginning of your symptoms did you start taking antiviral medication?

- Same day (within 24 hours)
- 1 day later
- 2 days later
- 3 days later
- 4 days later
- 5-7 days later
- More than 7 days later
- I don’t know/can’t remember

**Weekly Q10 (if symptoms)**
Did you change your daily routine because of your illness?

• No

• Yes, but I did not take time off work/school

• Yes, I took time off work/school

**Weekly Q10b (if symptoms) & (if taken time off work/school):**

Are you still off work/school?

• Yes

• No

• Other (e.g. I wouldn’t usually be at work/school today anyway)

**Weekly Q10c (if symptoms) & (if taken time off work/school):**

How have you been off work/school for?

• 1day

• 2days

• 3days

• 4days

• 5days

• 6to10days

• 11 to 15 days

• More than 15 days

**Weekly Q11 (if symptoms)**
What do you think is causing your symptoms?

- Flu or flu-like illness o Common cold
- Allergy/hay fever
- Asthma
- Gastroenteritis/gastric flu
- Other
- I don’t know
