# Supplementary material for: Participatory Online Surveillance as a Supplementary Tool to Sentinel Doctors for Influenza-Like Illness Surveillance in Italy
Source: PLoS One. 2017 Jan 11;12(1):e0169801. doi: 10.1371/journal.pone.0169801 (PMC5226807; doi:10.1371/journal.pone.0169801)
Supplement: S2 File — (DOCX) [file pone.0169801.s002.docx]

# **Supporting Information**


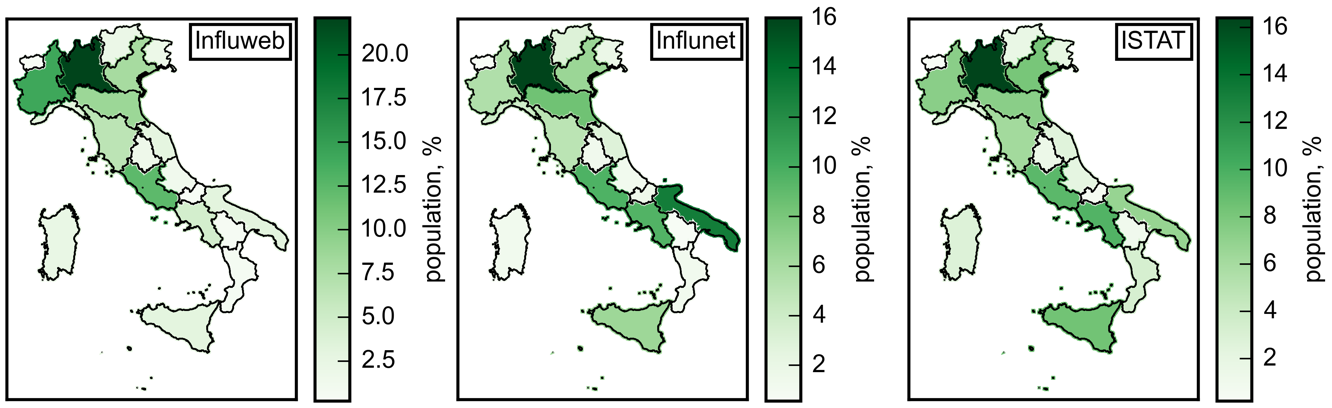


**A Fig.** Geographical distribution of the Influweb active participants in comparison with the Influnet sample and the Italian population for the 2012-2013 influenza season.


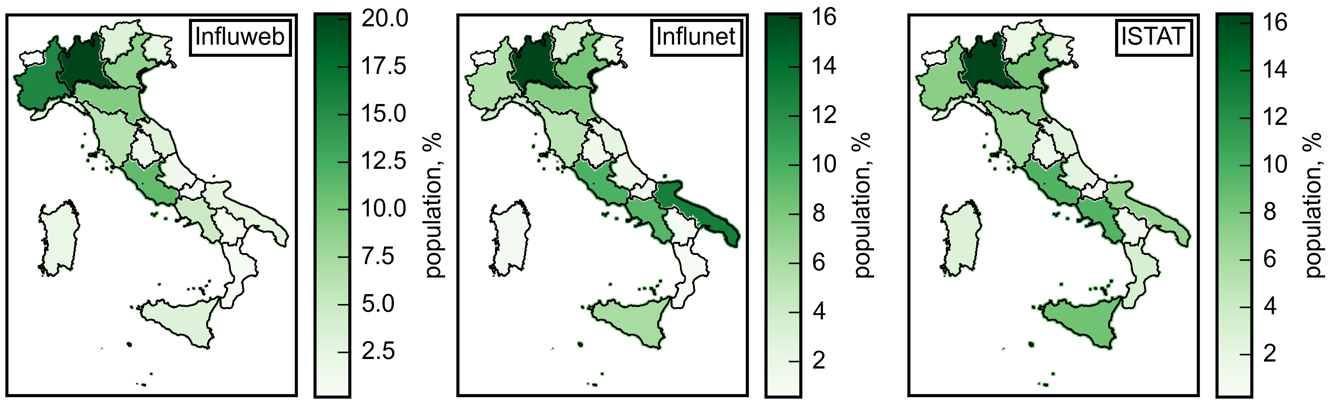


**B Fig.** Geographical distribution of the Influweb active participants in comparison with the Influnet sample and the Italian population for the 2013-2014 influenza seasons.


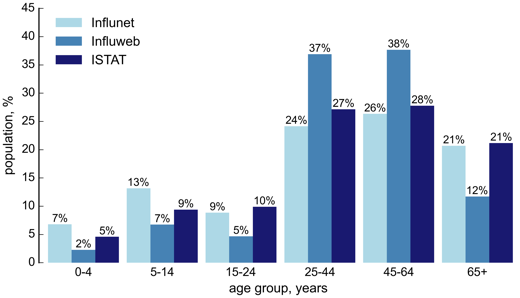

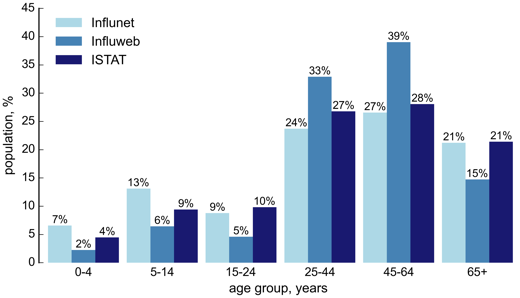

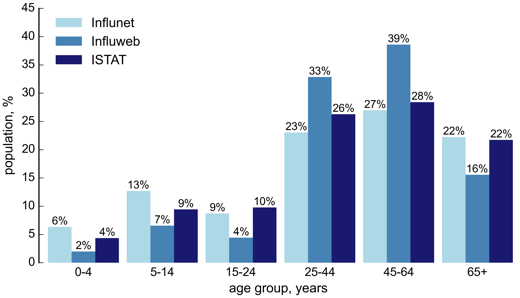


**C Fig.** Comparison of the Influweb population with the Influnet sample and the Italian general population for the 2012-2013, 2013-2014 and 2014-2015 respectively.


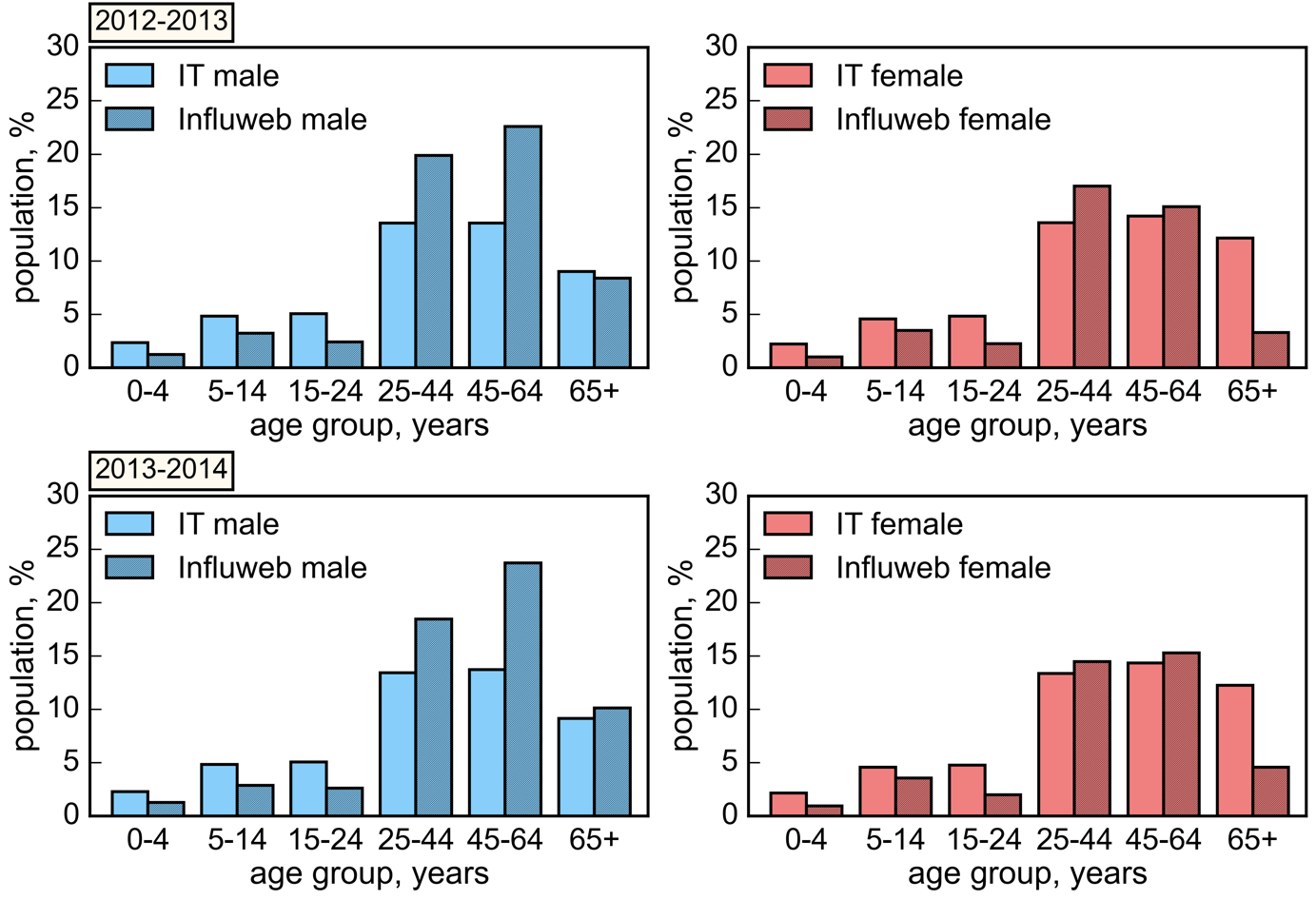


**D Fig.** Comparison of the Influweb population in different age and gender categories with the Italian population for the three seasons under study.

| **season** | **baseline weeks** | **Estimated Attack Rate % (95% CI)** | | |
| --- | --- | --- | --- | --- |
|  |  | **Age Groups, years** | | |
|  |  | **25-44** | **45-64** | **≥ 65** |
| 2012-2013 | 47-51 | 16.06 (0, 39.92) | 18.72 (0, 38.95) | - |
|  | 46-51 | 10.76 (0, 35.49) | 18.7 (0, 38.96) | - |
|  | 46-50 | 8.89 (0, 35.67) | 12.76 (0, 35.59) | - |
|  | 47-50 | 15.7 (0, 41.15) | 12.77 (0, 35.56) | - |
| 2013-2014 | 47-52 | 10.75 (0, 31.3) | 4.04 (0, 18.42) | 12.04 (0, 28.4) |
|  | 46-52 | 10.8 (0, 30.72) | 6.63 (0, 20.17) | 13.0 (0, 28.92) |
|  | 47-51 | 12.47 (0, 33.57) | 4.48 (0, 19.35) | 10.5 (0, 27.56) |
|  | 46-51 | 11.05 (0, 31.77) | 4.99 (0, 19.27) | 11.65 (0, 28.17) |
| 2014-2015 | 47-51 | 16.2 (0, 41.39) | 19.43 (0, 38.73) | 15.32 (0, 38.72) |
|  | 46-51 | 19.23 (0, 42.41) | 17.51 (0, 36.22) | 19.18 (0, 41.48) |
|  | 46-50 | 17.82 (0, 42.3) | 16.66 (0, 36.43) | 24.3 (0.63, 45.98) |
|  | 47-50 | 12.37 (0, 39.02) | 20.14 (0, 40.3) | 21.46 (0, 44.53) |

**A Table.** Sensitivity analysis with respect to the choice of the baseline period for the attack rates analysis.
